# Complete motor recovery after acute paraparesis caused by spontaneous spinal epidural hematoma: case report

**DOI:** 10.1186/1471-227X-11-10

**Published:** 2011-07-27

**Authors:** Leandro U Taniguchi, Felix H Pahl, José ED Lúcio, Roger S Brock, Marcos QT Gomes, Tarso Adoni, Victor CC Fiorini, Rodrigo C Carvalho, Eli F Evaristo, Eduardo G Mutarelli, Guilherme Schettino

**Affiliations:** 1 Discipline of Emergency Medicine, Faculdade de Medicina da Universidade de São Paulo, Av. Dr. Enéas de Carvalho Aguiar 255, sala 5023, São Paulo, Brazil; 2 Hospital Sírio Libanês, Rua Dona Adma Jafet 91, São Paulo, Brazil; 3 Department of Neurology, Faculdade de Medicina da Universidade de São Paulo, Av. Dr. Enéas de Carvalho Aguiar 255, sala 5084, São Paulo, Brazil

## Abstract

**Background:**

Spontaneous spinal epidural hematoma is a relatively rare but potentially disabling disease. Prompt timely surgical management may promote recovery even in severe cases.

**Case presentation:**

We report a 34-year-old man with a 2-hour history of sudden severe back pain, followed by weakness and numbness over the bilateral lower limbs, progressing to intense paraparesis and anesthesia. A spinal magnetic resonance imaging scan was performed and revealed an anterior epidural hematoma of the thoracic spine. He underwent an emergency decompression laminectomy of the thoracic spine and hematoma evacuation. Just after surgery, his lower extremity movements improved. After 1 week, there was no residual weakness and ambulation without assistance was resumed, with residual paresthesia on the plantar face of both feet. After 5 months, no residual symptoms persisted.

**Conclusions:**

The diagnosis of spontaneous spinal epidural hematoma must be kept in mind in cases of sudden back pain with symptoms of spinal cord compression. Early recognition, accurate diagnosis and prompt surgical treatment may result in significant improvement even in severe cases.

## Background

Spontaneous spinal epidural hematoma (SSEH) is a rare cause of back pain in the emergency department (estimated incidence of approximately 0.1 per 100,000 patients per year [1]) but one that carries high morbidity. The classic clinical presentation is acute onset of severe, often radiating, back pain followed by signs and symptoms of nerve root and/or spinal cord compression, which develops minutes to days later [2-4]. The true etiology of SSEH remains unknown, but associations with some predisposing conditions, such as coagulopathies, blood dyscrasias and arteriovenous malformation, have been reported [5,6]. Although there are occasional reports of nonoperative treatments, timely surgical extirpation of the epidural clot remains the standard management [7].

This article presents a previously healthy young man who was admitted to the emergency department with back pain and symptoms of spinal cord compression caused by SSEH, in whom prompt surgical treatment prevented definitive neurological sequelae.

### Case presentation

A 34-year-old man presented to the emergency department with a 2-hour history of sudden acute severe back pain at the thoracic level. He described that the pain was initially dull and then became sharp. There was no history of trauma, drug use or any physical exertion. The past medical history was unremarkable. On arrival, he was conscious and alert, with no respiratory distress and normal vital signs. The rest of the physical examination was normal. Initial laboratory tests including complete blood count, chemistry panel and coagulation evaluation revealed no remarkable contributions.

During observation, at 3 hours from the beginning of the pain, the patient complained of weakness and numbness over the lower limbs. A physical examination revealed paraparesis (muscle power scores of 2/5 for thigh flexion muscles and 2/5 for foot flexion on the bilateral lower limbs) with flaccid lower extremities and bilateral extensor plantar responses, decreased sensation below the T10 dermatome with anesthesia in the perineal region, and urine retention. Neurological and neurosurgical consultants were contacted and emergency magnetic resonance imaging (MRI) was ordered.

**Figure 1:**
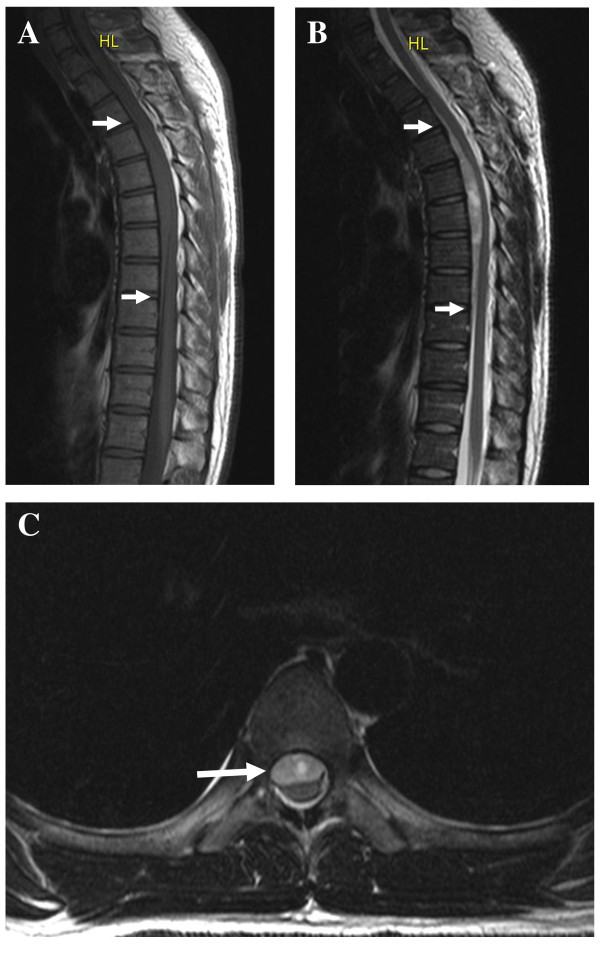
**Magnetic resonance imaging of the thoracic spine**. A, Sagittal T1-weighted imaging revealed an isointense anterior epidural mass extending from T3-T4 to T8-T9 (white arrows at the extremities of the mass), which compressed the spinal cord posteriorly, especially at the T5-T6 level. B, Sagittal T2-weighted imaging was helpful for delineation of the mass (white arrows at the extremities of the mass), which showed hyperintensity in this scan. The length was the same as that visualized with T1-weighted imaging, but the severity of the spinal cord compression was better visualized. C, Axial T2-weighted imaging revealed the epidural mass (white arrow) located in the anterior aspect of the spinal canal, compressing the spinal cord and obliterating the liquoric space.

MRI of the thoracic spine demonstrated an anterior epidural mass extending from T3-T4 to T8-T9, causing spinal cord compression, especially at T5-T6. The mass had isointensity to the spinal cord on T1-weighted images and hyperintensity on T2-weighted images (Figure 1). Diffusion-weighted imaging revealed no alterations in the spinal cord. Based on the clinical presentation and imaging findings, an epidural hematoma of the thoracic spine was suspected. The patient was administered 1 g of methylprednisolone intravenously and was taken to the operating room for an emergency decompression laminectomy at approximately 130 minutes after the initial onset of the spinal cord compression symptoms. A bilateral laminectomy from T5 to T7 was performed. During the operation, an epidural hematoma was discovered and evacuated. The pathologic report described a hematoma without neoplasm or vessel malformation. Postoperative angiography showed no vascular malformation.

Just after recovery from the anesthesia, the patient was able to lift both legs against gravity for some seconds. After 1 week, he could walk without assistance and had full strength in both legs. The patient regained sensation almost completely, although hypoesthesia remained at the soles of both feet and some perineal areas. Joint position and vibration sense were normal in the lower limbs. The patient also exhibited urinary retention requiring self-catheterization and constipation requiring medications for 1 month after surgery. After 5 months, the patient had recovered completely, with no residual symptoms.

## Conclusion

SSEH is defined as accumulation of blood in the vertebral epidural space that has no obvious cause. It represents 40% of all spinal epidural hematomas [8,9]. The pathogenesis is unclear but the bleeding is assumed to be of venous origin [6]. The valveless epidural venous plexus is particularly vulnerable to variations in pressure from the abdominal and thoracic cavities [6,8]. Hematomas are usually located posterior to the spinal cord, which is consistent with the anatomical location of the venous plexus [9,10]. In one large literature survey of case reports of spinal hematomas of any causes, Kreppel et al. [9] described that almost 75% of spinal hematomas are located posterior to the spinal cord. Ventral hematomas, as in our case, represented only 5% of all cases. Other authors have also described this posterior predominance [7,10,11].

SSEH occurs in all age groups, but most frequently after the fourth decade of life [10]. The most common localizations in adults are the cervicothoracic and thoracolumbar junctions [6]. The clinical presentation in most cases is the same as in our case, namely sudden-onset back pain followed by signs of nerve root or spinal cord compression. The symptoms of spinal cord compression may include ascending numbness, progressive paraplegia and/or loss of leg sensory function, and cauda-equina syndrome [1,11]. However, owing to its rarity, the exact diagnosis of SSEH may be difficult in a timely manner. The differential diagnosis includes spinal abscess, tumor, ischemia, transverse myelitis and acute vertebral disc disease [6].

Since the results of operative decompression of the spinal cord depend on the duration of the symptoms, time lost during diagnostic procedures may have negative influences on the outcome [7,9-13]. Consequently, accurate neuroradiologic confirmation of the correct diagnosis is mandatory. In the past, lumbar myelography and computed tomography scanning were used for diagnosis. However, these techniques are nonspecific, may not provide the accurate length of the hematoma and may produce false-negative findings [11,14]. Currently, spinal MRI has replaced these techniques as the initial diagnostic tool for SSEH. MRI is noninvasive, accurate and can demonstrate the localization and length of the hematoma as well as the effects on the spinal cord [1,10,11]. Furthermore, on T2-weighted images, hyperintense signals in the compressed spinal cord, suggesting intramedullary edema, may portend poor neurological recovery [10]. In our case, MRI provided detailed information about the magnitude, localization, dimension, limits and nature of the epidural mass. Although the compression of the spinal cord was significant (Figure 1C), there were no hyperintense signals on T2-weighted images of the spinal cord. This was correlated with a good postoperative recovery, in much the same way as described above [10].

The most relevant aspect of this case report is the early surgical management. This factor may have been the crucial determinant of the good neurologic outcome in our case. Many authors have already described that the speed of surgical intervention is correlated with better neurological and functional recovery [7-12]. A time frame of less than 12 hours from the initial ictus seems to be the best therapeutic window [10-12]. In our case, the patient underwent surgery at slightly more than 2 hours after the onset of the symptoms of spinal cord compression, and long before any neurological structural damage could be identified by MRI. Therefore, based in our case and the literature reviewed, we emphasize that SSEH is a neurosurgical emergency requiring immediate surgical intervention.

Another factor besides surgical timing that might affect the outcome is the patient's preoperative neurological status. Groen et al. [7] expertly reviewed the literature and reported 330 cases of SSEH. In their paper, postoperative recovery was correlated with sensorimotor impairment before surgery. Other authors described similar results in smaller case series [10,12]. Of note, complete loss of sensorimotor function may be recovered after decompressive laminectomy [7,13]. Other factors such as age, sex, and size and position of the hematoma were not correlated with the postoperative outcome [7]. Spinal cord infarction after decompressive laminectomy may also complicate the postoperative course and impair recovery [15].

In conclusion, although SSEH is rare in the emergency department, it is a critical diagnosis to consider in cases of sudden back pain with symptoms of spinal cord compression. Urgent spinal MRI is crucial for correct diagnosis, and decompressive surgical management with evacuation of the hematoma is imperative. Fast and solid clinical recognition and diagnosis combined with appropriate treatment may improve the neurological and functional outcomes.

## Consent

Written informed consent was obtained from the patient's next-of-kin for publication of this case report and any accompanying images. A copy of the written consent is available for review by the Editor-in-Chief of this journal.

## Competing interests

The authors declare that they have no competing interests.

## Authors' contributions

LUT...Manuscript preparation, Literature search, Data collection, Data interpretation. FHP...Chief Neurosurgeon who operated on the patient, Data collection, Manuscript preparation. JEDL...Neurosurgeon who operated on the patient, Data collection, Manuscript preparation. RSB...Neurosurgeon who operated on the patient, Data collection, Manuscript preparation. MQTG...Neurosurgeon who operated on the patient, Data collection, Manuscript preparation. TA...Neurologist who treated the patient, Data collection, Manuscript preparation. VCCF...Neurologist who treated the patient, Data collection, Manuscript preparation. RCC...Neurologist who treated the patient, Data collection, Manuscript preparation. EFE...Neurologist who treated the patient, Data collection, Manuscript preparation. EGM...Chief Neurologist who treated the patient, Data collection, Manuscript preparation. GS...Manuscript preparation, Literature search. All authors read and approved the final manuscript.

## Pre-publication history

The pre-publication history for this paper can be accessed here:

http://www.biomedcentral.com/1471-227X/11/10/prepub

## References

[B1] Holtas S Heiling M Lönntoft M Spontaneous spinal epidural hematoma: findings at MR imaging and clinical correlation Radiology 1996 199 409 413 8668786 10.1148/radiology.199.2.8668786

[B2] Liu WH Hsieh CT Chiang YH Chen GJ Spontaneous spinal epidural hematoma of thoracic spine: a rare case report and review of literature Am J Emerg Med 2008 26 384.e1 384.e2 10.1016/j.ajem.2007.05.036 18358974

[B3] Lannum S Stratton J Spontaneous epidural hematoma of the thoracic spine in a 17-year-old adolescent boy: a case report Am J Emerg Med 2009 27 628.e5 628.e6 10.1016/j.ajem.2008.08.031 19497479

[B4] Miller JB Khalsa G Vohra T Spontaneous spinal epidural hematoma presenting as flank pain and constipation Am J Emerg Med 2010 28 536.e3 536.e5 10.1016/j.ajem.2009.04.018 20466256

[B5] Dinsmore AJ Leonard RB Manthey D Spontaneous spinal epidural hematoma: a case report J Emerg Med 2005 28 423 426 10.1016/j.jemermed.2004.11.023 15837023

[B6] Groen RJ Ponssen H The spontaneous spinal epidural hematoma. A study of the etiology J Neurol Sci 1990 98 121 138 2243224 10.1016/0022-510x(90)90253-j

[B7] Groen RJ van Alphen HA Operative treatment of spontaneous spinal epidural hematomas: a study of the factors determining postoperative outcome Neurosurgery 1996 39 494 509 8875479 10.1097/00006123-199609000-00012

[B8] Patel H Boaz JC Phillips JP Garg BP Spontaneous spinal epidural hematoma in children Pediatr Neurol 1998 19 302 307 10.1016/S0887-8994(98)00059-9 9831003

[B9] Kreppel D Antoniadis G Seeling W Spinal hematoma: a literature survey with meta-analysis of 613 patients Neurosurg Rev 2003 26 1 49 10.1007/s10143-002-0224-y 12520314

[B10] Liao CC Lee ST Hsu WC Chen LR Lui TN Lee SC Experience in the surgical management of spontaneous spinal epidural hematoma J Neurosurg 2004 100 1 Suppl Spine 38 45 14748572 10.3171/spi.2004.100.1.0038

[B11] Alexiadou-Rudolf C Ernestus RI Nanassis K Lanfermann H Klug N Acute nontraumatic spinal epidural hematomas: an important differential diagnosis in spinal emergencies Spine 1998 23 1810 1813 10.1097/00007632-199808150-00018 9728384

[B12] Lawton MT Porter RW Heiserman JE Jacobowitz R Sonntag VKH Dickman CA Surgical management of spinal epidural hematoma: relationship between surgical timing and neurological outcome J Neurosurg 1995 83 1 7 10.3171/jns.1995.83.1.0001 7782824

[B13] McQuarrie IG Recovery from paraplegia caused by spontaneous spinal epidural hematoma Neurology 1978 28 224 228 564475 10.1212/wnl.28.3.224

[B14] Avrahami E Tadmor R Ram Z Feibel M Itzhak Y MR demonstration of spontaneous acute epidural hematoma of the thoracic spine Neuroradiology 1989 31 89 92 2717013 10.1007/BF00342039

[B15] Park J Lee JB Park JY Lim DJ Kim SD Chung YK Spinal cord infarction after decompressive laminectomy for spontaneous spinal epidural hematoma Neurol Med Chir (Tokyo) 2007 47 325 327 10.2176/nmc.47.325 17652921

